# Impact of the 2017 ACC/AHA hypertension guidelines on antihypertensive prescribing in the United States: real-world evidence from a nationally representative population

**DOI:** 10.3389/fphar.2026.1787466

**Published:** 2026-02-25

**Authors:** Eissa A. Jafari

**Affiliations:** 1 Department of Pharmacy Practice, College of Pharmacy, Jazan University, Jazan, Saudi Arabia; 2 Pharmacy Practice Research Unit, College of Pharmacy, Jazan University, Jazan, Saudi Arabia

**Keywords:** 2017 ACC/AHA hypertension guidelines, antihypertensive, fixed-dose combination antihypertensives, hypertension, prescribing

## Abstract

**Background:**

Hypertension (HTN) remains a major contributor to cardiovascular morbidity and mortality in the United States (US). The 2017 ACC/AHA HTN guidelines introduced major changes to diagnostic thresholds and treatment recommendations, including earlier pharmacological initiation and greater emphasis on combination therapy. However, the long-term, population-level impact of these guidelines on antihypertensive medication utilization in the US remains uncharacterized.

**Methods:**

We conducted a pooled cross-sectional study using data from the Medical Expenditure Panel Survey (2013–2022). Adults ≥18 years with diagnosed HTN were included. Antihypertensive classes utilization was defined as any use of the medication class with ≥2 prescription refills within the same year among eligible participants. Utilization of antihypertensive classes was then pooled and examined across two periods: pre-guidelines (2013–2017) and post-guidelines (2018–2022). Survey-weighted multivariable logistic regression models were used to assess the impact of the 2017 ACC/AHA guidelines on the overall utilization of antihypertensive drug classes and within subgroups with compelling indications.

**Results:**

A total of 29,901 adults were included. Following guidelines implementation, angiotensin receptor blockers (ARBs) utilization increased from 18% to 26% (adjusted OR [aOR] = 1.35; 95% confidence interval [CI]: 1.21–1.50, p < 0.0001), and calcium channel blocker (CCB) use increased from 28% to 32% (aOR = 1.24; 95% CI: 1.13–1.36, p < 0.0001). In contrast, fixed-dose combination (FDC) utilization declined from 22% to 16% (aOR = 0.67; 95% CI: 0.59–0.75, p < 0.0001). Utilization of other antihypertensive classes did not change significantly.

**Conclusion:**

After the 2017 ACC/AHA guidelines update, antihypertensive prescribing in the US showed increased use of ARBs and CCBs. However, declining FDC use highlights a persistent gap between evidence-based guidance and real-world practice.

## 1 Introduction

Hypertension (HTN) remains the most prevalent chronic condition among adults in the United States (US) and a leading modifiable risk factor for cardiovascular morbidity and mortality worldwide (Mills et al., 2020; Dorans et al., 2018). It accounts for nearly half of all cardiovascular events, including myocardial infarction and stroke, and contributes to more than 10 million deaths annually (Mills et al., 2020; Wu et al., 2025). Despite decades of public health efforts and the availability of effective, affordable pharmacological therapies, HTN control remains suboptimal. Recent National Health and Nutrition Examination Survey data (2021–2023) indicated that nearly half of US adults meet diagnostic criteria for HTN (≥130/80 mm Hg), yet fewer than half achieve adequate blood pressure (BP) control (Fryar et al., 2023). Therapeutic inertia and suboptimal medication utilization continue to undermine effective HTN management, reinforcing the need to optimize antihypertensive treatment strategies to reduce adverse cardiovascular outcomes (Zhou et al., 2021).

HTN management in the US has evolved through successive guidelines updates informed by emerging clinical evidence and expert consensus (Chobanian et al., 2003; James et al., 2014; Whelton et al., 2017). The Seventh Report of the Joint National Committee (JNC 7), published in 2003, defined HTN using a threshold of ≥140/90 mm Hg and emphasized lifestyle modification alongside stepwise pharmacological therapy, recommending thiazide diuretic as first-line treatment for most patients and specific drug classes for compelling indications (Chobanian et al., 2003). In 2014, the Eighth Joint National Committee (JNC 8) retained the diagnostic threshold of ≥140/90 mm Hg but relaxed BP targets for adults aged ≥60 years to <150/90 mm Hg. JNC 8 also expanded first-line treatment options to include thiazide diuretic, angiotensin-converting enzyme inhibitors (ACEI), angiotensin receptor blocker (ARB), and calcium channel blocker (CCB) (James et al., 2014). Additionally, Beta-blocker (BB) were no longer recommended as initial therapy owing to inferior stroke and composite cardiovascular outcomes, compared with other classes (James et al., 2014). Although JNC 8 offered greater therapeutic flexibility, it was criticized for potentially reducing treatment intensity in high-risk older adults. Evidence of suboptimal BP control and of rising cardiovascular events under the JNC 8 targets prompted renewed emphasis on risk-stratified, earlier pharmacologic intervention (James et al., 2014; Shrout et al., 2017).

A major shift in HTN management was introduced by the 2017 American College of Cardiology (ACC)/American Heart Association (AHA) HTN guidelines, which redefined HTN as BP ≥ 130/80 mm Hg, expanding the treatment eligibility (Whelton et al., 2017; Muntner et al., 2018). By lowering the BP threshold for HTN diagnosis and recommending pharmacotherapy for stage 1 HTN with elevated atherosclerotic cardiovascular disease risk, the 2017 guideline increased the number of adults eligible for antihypertensive treatment (Whelton et al., 2017; Muntner et al., 2018). Additionally, the new guidelines recommended initiating two antihypertensive agents, preferably as a fixed-dose combination (FDC), when baseline BP exceeded the target by ≥ 20/10 mm Hg (James et al., 2014; Whelton et al., 2017). The 2017 update also standardized the target BP goal (<130/80 mm Hg) across adult populations, including those with diabetes and chronic kidney disease (CKD). The 2017 ACC/AHA guidelines recommended thiazide diuretic, ACEI, ARB, and CCB as first-line options, and discouraged the use of beta blockers outside specific compelling indications, such as heart failure or post-myocardial infarction (Whelton et al., 2017).

Despite these substantial changes, the real-world impact of the 2017 ACC/AHA guidelines on antihypertensive prescribing patterns remains poorly characterized (Muntner et al., 2018). Most post-guidelines studies have focused on HTN prevalence, control, or treatment eligibility, rather than on temporal changes in medication utilization (Dorans et al., 2018; Muntner et al., 2018; Khera et al., 2018; Hussain et al., 2022). The existing evidence on the impact of the 2017 ACC/AHA guidelines on antihypertensive utilization relied on data up to 2019, limiting the ability to capture long-term antihypertensive prescribing trends (Lin et al., 2024). Moreover, little is known about whether the guidelines implementation had a differential impact on high-risk populations, such as Non-Hispanic Black adults and those with compelling indications.

To address this gap, this study aimed to evaluate the impact of the 2017 ACC/AHA HTN guidelines on antihypertensive medication utilization, using nationally representative data from the Medical Expenditure Panel Survey (MEPS) from 2013 through 2022. Antihypertensive prescribing patterns were examined over the 5 years preceding (2013–2017) and 5 years following (2018–2022) guidelines publication. By leveraging a decade of population-level data, this study provided contemporary, real-world evidence on how major HTN guidelines influenced antihypertensive medication use, with implications for clinical practice, health policy, and strategies to improve HTN control in the US.

## 2 Methods

### 2.1 Data source

This study used data from the Household Component of the MEPS (MEPS-HC) for the years 2013 through 2022. MEPS is a nationally representative survey of the U.S. civilian, noninstitutionalized population conducted annually by the Agency for Healthcare Research and Quality. It employs a stratified, multistage probability sampling design and collects detailed information on sociodemographic characteristics, medical conditions, prescribed medications, healthcare utilization, expenditures, and insurance coverage.

Data are collected through a series of in-person household interviews and are supplemented by the Medical Provider Component, which verifies information from healthcare providers, hospitals, and pharmacies, and the Insurance Component, which captures details on employer-sponsored healthcare plans. The MEPS sample is drawn from respondents to the National Health Interview Survey, ensuring national representativeness.

For this study, we used the full-year consolidated file, the prescribed medicines file, and the medical conditions file from the MEPS-HC. These files were linked using the unique person identifier (DUPERSID). The full-year consolidated file provided demographic, socioeconomic, behavioral, and health-related variables; the prescribed medicines file contained detailed information on dispensed medications; and the medical conditions file included diagnosis information.

All MEPS data are publicly available and fully de-identified, collected under the authority of the Public Health Service Act. Because this analysis used publicly available secondary data, it was considered exempt from the institutional review board and did not require informed consent.

### 2.2 Study design and population

This was a pooled cross-sectional study. Adults aged ≥18 years were eligible if they had a diagnosis of HTN identified using International Classification of Diseases, Ninth or Tenth Revision (ICD-9/10) codes or self-reported HTN and had ≥2 prescription refills for any antihypertensive medication during the same year. The ≥2 refill criterion was applied to reduce treatment misclassification and increase the likelihood of capturing prescriptions that represent ongoing antihypertensive therapy rather than a single trial prescription, or acute use. Antihypertensive medications were identified using Multum Lexicon therapeutic classification codes (Supplementary Table S1) from the prescribed medicines file. Individuals with a pregnancy diagnosis during the study period were excluded.

### 2.3 Study outcomes

The study outcome was the annual utilization of each antihypertensive medication class, assessed within each calendar year. Annual person-year utilizations were then pooled into two multi-year periods: pre-guidelines (2013–2017) and post-guidelines (2018–2022), corresponding to periods before and after publication of the 2017 ACC/AHA HTN guidelines. Antihypertensive classes included in the study were ACEI, ARB, CCB, diuretic, BB, central-acting alpha agonist (CAA), alpha-1 peripherally acting antagonist (APA) so it matches the Figure 2, aldosterone receptor antagonist (ARA), vasodilator, and FDC product.

### 2.4 Covariates

The primary exposure variable was the time period, defined as pre-guidelines (2013–2017) vs. post-guidelines (2018–2022). Other variables included age, sex (male/female), Race/ethnicity (Non-Hispanic White, Non-Hispanic Black, Hispanic, Asian, Other), education level (no degree, high school diploma, some college or associate degree, bachelor’s or higher degree), health insurance coverage (private, public, uninsured), census region (Northeast, Midwest, South, West), poverty category (poor/negative, near poor, low income, middle income, high income), marital status (married; never married; divorced, widowed, or separated), and physical exercise.

Clinical comorbidities included diabetes, CKD, heart failure, coronary heart disease, myocardial infarction, stroke, dyslipidemia, chronic obstructive pulmonary disorder (COPD), asthma, Alzheimer’s disease and related dementias (ADRD), osteoarthritis, gastroesophageal reflux disorder (GERD), anxiety, and depression.

### 2.5 Statistical analysis

All analyses accounted for the complex MEPS survey design by applying person-level weights, strata, and primary sampling units to produce nationally representative estimates. Descriptive statistics were used to summarize participant characteristics, reported as frequency and weighted percentage (wt%) for categorical variables and weighted means, with standard error for continuous variables. Differences between the pre- and post-guidelines periods were evaluated using the Chi-square test for categorical variables and t-tests for continuous variables.

Survey-weighted logistic regression models were fitted to estimate the odds of utilization of each antihypertensive medication class in the post-guidelines period compared with the pre-guidelines period. Each medication class was modeled as a binary outcome, with the guidelines period as the primary independent variable. Models were adjusted for demographic, socioeconomic, behavioral, and clinically relevant covariates. Results were reported as odds ratios (ORs) with 95% confidence intervals (CIs) and p-values.

To further assess adherence to 2017 ACC/AHA recommendations among patients with compelling indications (e.g., diabetes, CKD, Black race), additional survey-weighted logistic regression models were performed using each indication as a subgroup, with antihypertensive class as the dependent variable and guidelines period as the independent variable. Given multiple subgroup-by-class comparisons, we applied the Benjamini–Hochberg procedure to control for the false discovery rate (FDR) at 5%. Associations were considered statistically significant if the q value < 0.05. All analyses were conducted using SAS 9.4 (SAS Institute, Cary, NC) and R version 4.3.3 (R Foundation for Statistical Computing, Vienna, Austria).

## 3 Results

### 3.1 Data preparation and cohort selection

**FIGURE 1:**
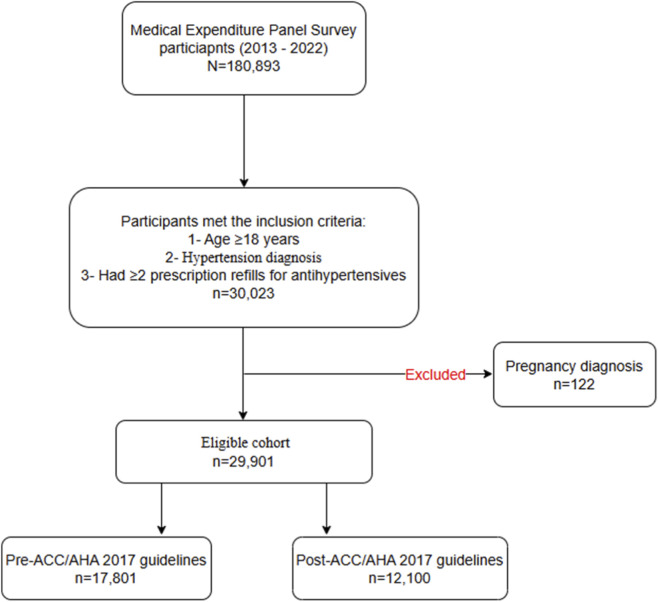
Data preparation and cohort selection. Abbreviation: ACC, American College of Cardiology; AHA, American Heart Association.

A total of 180,893 participants from the 2013–2022 MEPS sample were screened for eligibility. Of these, 30,023 adults met the inclusion criteria of being aged ≥18 years, diagnosed with HTN, and having at least two refills of prescribed antihypertensive medications. After excluding 122 pregnant participants, the final analytic cohort comprised of 29,901 adults with treated HTN, including 17,801 participants in the pre-2017 ACC/AHA guidelines period and 12,100 in the post-2017 ACC/AHA guidelines period (Figure 1).

### 3.2 Patient characteristics

Demographic, socioeconomic, and clinical characteristics of the study population overall and by guidelines period are presented in Table 1. The cohort mean age was 63 years, with an equal distribution of males and females. The cohort was predominantly non-Hispanic White (69%), followed by non-Hispanic Black (14%), Hispanic (10%), non-Hispanic Asian (4%), and other races (3%). The majority of participants had at least a high school education (45%), private health insurance (62%), high income status (42%), and were married (58%). The most prevalent comorbidities were dyslipidemia (68%), diabetes (30%), osteoarthritis (30%), GERD (18%), coronary heart disease (16%), and anxiety (16%) (Table 1).

**TABLE 1:** Patient characteristics.

Characteristics	Overall N = 29,901 (wt%)	Pre-2017 ACC/AHA Guidelines n = 17,801 (wt%)	Post-2017 ACC/AHA Guidelines n = 12,100 (wt%)	P-value
Age	63 (0.14)	63 (0.18)	66 (0.20)	0.0019
Age category	​	​	​	0.0053
18–39	1,336 (5)	907 (5)	429 (5)	​
40–64	13,332 (45)	8,594 (46)	4,738 (43)	​
≥65	15,233 (50)	8,300 (49)	6,933 (52)	​
Sex	​	​	​	0.0142
Female	13,700 (50)	8,058 (49)	5,642 (51)	​
Male	16,201 (50)	9,743 (51)	6,458 (49)	​
Race/Ethnicity	​	​	​	0.2751
Hispanic	4,783 (10)	3,258 (10)	1,525 (10)	​
Non-hispanic white	16,236 (69)	8,572 (69)	7,664 (69)	​
Non-hispanic back	6,573 (14)	4,471 (14)	2,102 (13)	​
Non-hispanic Asian	1,500 (4)	1,046 (4)	454 (5)	​
Other or multiple races	809 (3)	454 (3)	355 (3)	​
Education	​	​	​	<0.0001
No degree	4,929 (12)	3,201 (14)	1,728 (10)	​
High school diploma	11,903 (45)	6,217 (43)	5,686 (47)	​
Some college/associate degree	3,633 (15)	2,404 (18)	1,229 (12)	​
Bachelor’s/higher education	6,414 (28)	3,016 (25)	3,398 (31)	​
Insurance coverage	​	​	​	<0.0001
Private	16,070 (62)	9,649 (64)	6,421 (60)	​
Public	12,636 (35)	7,203 (33)	5,433 (38)	​
Uninsured	1,195 (3)	949 (3)	246 (2)	​
Poverty category	​	​	​	0.0003
Poor/negative	5,271 (11)	3,236 (12)	2,035 (11)	​
Near poor	1,846 (5)	1,179 (5)	667 (4)	​
Low income	4,663 (14)	2,901 (14)	1,762 (13)	​
Middle income	8,245 (28)	5,000 (28)	3,245 (28)	​
High income	9,876 (42)	5,485 (41)	4,391 (44)	​
Marital status	​	​	​	0.2369
Married	15,311 (58)	9,173 (57)	6,138 (59)	​
Divorced/widowed/separated	10,947 (32)	6,386 (33)	4,561 (31)	​
Never married	3,643 (10)	2,242 (10)	1,401 (10)	​
Region	​	​	​	0.7012
Northeast	4,899 (17)	2,978 (18)	1,921 (16)	​
Midwest	6,170 (22)	3,585 (22)	2,585 (22)	​
South	12,516 (41)	7,477 (41)	5,039 (42)	​
West	6,316 (19)	3,761 (19)	2,555 (19)	​
Physical exercise	12,251 (43)	7,006 (41)	5,245 (45)	<0.0001
Diabetes	9,924 (30)	6,169 (31)	3,755 (29)	0.0253
Chronic kidney disease	622 (2)	349 (2)	273 (2)	0.5721
Heart failure	876 (3)	539 (3)	337 (3)	0.0231
Coronary heart disease	4,848 (16)	2,977 (17)	1,871 (15)	<0.0001
Stroke	3,550 (11)	2,147 (11)	1,403 (11)	0.1263
Myocardial infarction	3,394 (11)	2,066 (12)	1,309 (10)	0.0051
Dyslipidemia	20,414 (68)	12,040 (68)	8,374 (68)	0.5040
COPD	3,993 (13)	2,563 (15)	1,430 (11)	<0.0001
Asthma	4,652 (15)	2,610 (14)	2,042 (16)	0.0004
ADRD	4,143 (12)	2,604 (13)	1,539 (11)	0.0015
Osteoarthritis	8,849 (30)	4,869 (29)	3,980 (31)	0.0587
GERD	5,340 (18)	3,173 (18)	2,167 (17)	0.0181
Anxiety	4,711 (16)	2,938 (18)	1,773 (14)	<0.0001
Depression	3,179 (11)	1,463 (8)	1,716 (14)	<0.0001

Abbreviation: HTN, hypertension; ACC, american college of cardiology; AHA, american heart association; COPD, chronic obstructive pulmonary disorder; GERD, gastroesophageal reflux disorder; ADRD, Alzheimer’s disease and related dementia; wt%, Weighted percentage.

Compared with participants in the pre-guidelines period, those in the post-guidelines period were older (66 vs 63 years, p = 0.0019), more likely to be female (51% vs 49%, p = 0.0142), had a higher proportion with a bachelor’s degree or higher education (31% vs 25%, p < 0.0001), and more high-income earners (44% vs 41%, p = 0.0003). In contrast, private insurance coverage was lower in the post-guidelines period (60% vs 64%, <0.0001), compared to those in the pre-guidelines period (Table 1).

Clinically, compared with those in post-guidelines period, the participants in pre-guidelines period had higher a prevalence of diabetes (31% vs 29%, p = 0.0253), coronary heart disease (17% vs 15%, p < 0.0001), myocardial infarction (12% vs 10%, p = 0.0051), COPD (15% vs 11%, p < 0.0001), ADRD (13% vs 11%, p = 0.0015), and anxiety (18% vs 14%, p < 0.0001). Conversely, asthma (16% vs 14%; p = 0.0004) and depression (14% vs 8%; p < 0.0001) were more prevalent in post-guidelines period participants, compared with those in the pre-guidelines period (Table 1).

### 3.3 Trends in antihypertensive use

**FIGURE 2:**
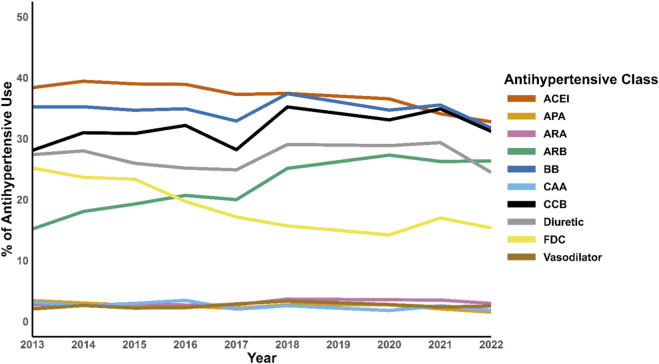
Trend of antihypertensive classes utilization before and after the 2017 ACC/AHA guidelines implementation. Abbreviation: ACC, American College of Cardiology; AHA, American Heart Association; ACEI, Angiotensin-converting enzyme inhibitors; ARB, Angiotensin receptor blocker; CCB, Calcium channel blocker, BB; Beta-blocker; CAA, Central alpha agonist; APA, alpha-1 peripherally acting antagonist; ARA, Aldosterone receptor antagonist; KSP, Potassium-sparing diuretics; FDC, Fixed-dose combination.

Antihypertensive medication utilization before and after implementation of the 2017 ACC/AHA guidelines is presented in Figure 2; Table 2. The most commonly prescribed drug classes were ACEI (38%), BB (35%), CCB (30%), and diuretic (26%).

**TABLE 2:** Antihypertensive classes utilization before and after the 2017 ACC/AHA guidelines implementation.

Antihypertensive class	Overall N = 29,901 (wt%)	Pre-2017 ACC/AHA Guidelines n = 17,801 (wt%)	Post-2017 ACC/AHA Guidelines n = 12,100 (wt%)	P-value
ACEI	11,187 (38)	6,863 (39)	4,324 (35)	<0.0001
ARB	6,381 (22)	3,240 (18)	3,141 (26)	<0.0001
CCB	9,418 (30)	5,302 (28)	4,116 (32)	<0.0001
Diuretics	8,110 (26)	4,699 (26)	3,411 (26)	0.4834
BB	10,462 (35)	6,169 (36)	4,293 (34)	0.0877
CAA	793 (2)	514 (3)	279 (2)	0.0191
APA	792 (3)	505 (3)	287 (2)	0.0018
ARA	911 (3)	489 (3)	422 (3)	0.0464
Vasodilator	772 (3)	421 (2)	351 (3)	0.0251
FDC	5,849 (19)	3,959 (22)	1,890 (16)	<0.0001

Abbreviation: ACC, american college of cardiology; AHA, american heart association; ACEI, Angiotensin-converting enzyme inhibitors; ARB, angiotensin receptor blocker; CCB, calcium channel blocker; BB, Beta-blocker; CAA, central alpha agonist; APA, alpha-1 peripherally acting antagonist; ARA, aldosterone receptor antagonist; KSP, Potassium-sparing diuretics; FDC, Fixed-dose combination; wt%, Weighted percentage.

Comparing pre- and post-guidelines periods, significant shifts in antihypertensives prescribing were observed. ARB use increased from 18% to 26% (p < 0.0001), and CCB increased from 28% to 32% (p < 0.0001). In contrast, ACEI use declined from 39% to 35% (p < 0.0001). FDC utilization also decreased from 22% to 16% (p < 0.0001) (Figure 2; Table 2).

**FIGURE 3:**
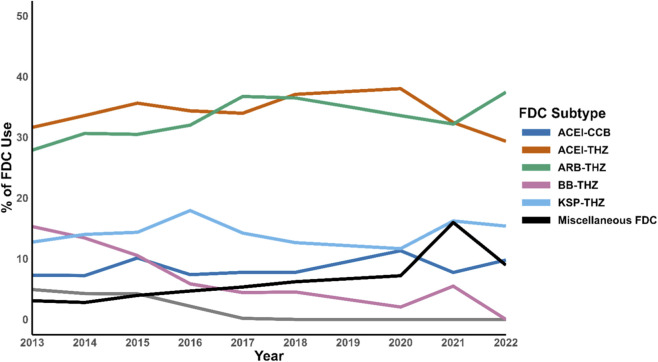
Trend of FDC utilization before and after the 2017 ACC/AHA guidelines implementation. Abbreviation: ACC, American College of Cardiology; AHA, American Heart Association; ACEI, Angiotensin-converting enzyme inhibitors; ARB, Angiotensin receptor blocker; THZ, thiazide diuretics; CCB, Calcium channel blocker; BB, Beta-blocker; KSP, Potassium-sparing diuretics; FDC, Fixed-dose combination.

Among FDC users, the most common combinations were ARB and thiazide diuretic (33%), ACEI and thiazide diuretic (33%), and potassium-sparing diuretic and thiazide diuretic (15%). While most FDC patterns remained relatively stable over time, BB and thiazide diuretic combination declined sharply (11%–3%; p < 0.0001), and miscellaneous FDC increased (3%–10%; p < 0.0001). No temporal changes were observed in dual renin–angiotensin system blockade combinations (Figure 3; Table 3).

**TABLE 3:** FDC utilization before and after the 2017 ACC/AHA guidelines implementation.

Antihypertensive class	Overall N = 5,849 (wt%)	Pre-2017 ACC/AHA Guidelines n = 3,959 (wt%)	Post-2017 ACC/AHA Guidelines n = 1,890 (wt%)	P-value
ACEI and thiazide diuretic	1,982 (33)	1,325 (32)	657 (34)	0.3676
ACEI and CCB	477 (8)	313 (8)	164 (8)	0.7088
ARB and thiazide diuretic	1,882 (33)	1,215 (32)	667 (35)	0.0894
BB and thiazide diuretic	506 (8)	440 (11)	66 (3)	<0.0001
ARB and CCB	143 (2)	143 (3)	0	​
KSP diuretic and thiazide diuretic	824 (15)	564 (15)	260 (14)	0.2745
Miscellaneous combination	318 (6)	149 (3)	169 (10)	<0.0001

Abbreviation: ACC, american college of cardiology; AHA, american heart association; ACEI, Angiotensin-converting enzyme inhibitors; ARB, angiotensin receptor blocker; CCB, calcium channel blocker; BB, Beta-blocker; KSP, Potassium-sparing diuretics; FDC, Fixed-dose combination; wt%, Weighted percentage.

### 3.4 Impact of 2017 ACC/AHA guidelines implementation on antihypertensive utilization

The results of multivariable survey-weighted logistic regression models assessing the impact of the 2017 ACC/AHA HTN guidelines on antihypertensive class utilization in the overall population are presented in Table 4. Compared with the pre-guidelines period, the post-guidelines period was associated with significantly higher odds of prescribing ARB (OR = 1.35, 95% CI: 1.21–1.50, p < 0.0001) and CCB (OR = 1.24, 95% CI: 1.13–1.36, p < 0.0001). In contrast, prescribing FDC was significantly lower post-guidelines (OR = 0.67, 95% CI: 0.59–0.75, p < 0.0001). No statistically significant changes were observed in ACEI, BB, diuretic, CAA, ARA, APA, or vasodilator utilization.

**TABLE 4:** Impact of 2017 ACC/AHA guidelines implementation on the antihypertensive classes utilization in the overall HTN population.

Antihypertensive class	Odds ratio of antihypertensive utilization post-2017 ACC/AHA guidelines	Lower 95% CI	Upper 95% CI	P-value
ACEI	0.97	0.88	1.07	0.5078
ARB	1.35	1.21	1.50	<0.0001
CCB	1.24	1.13	1.36	<0.0001
Diuretic	1.10	0.98	1.22	0.0963
BB	1.05	0.94	1.16	0.3957
CAA	0.85	0.67	1.07	0.1692
APA	0.93	0.74	1.18	0.5569
ARA	1.14	0.89	1.47	0.3063
Vasodilator	1.24	0.92	1.67	0.1645
FDC	0.67	0.59	0.75	<0.0001

Abbreviation: ACC, american college of cardiology; AHA, american heart association; ACEI, Angiotensin-converting enzyme inhibitors; ARB, angiotensin receptor blocker; CCB, calcium channel blocker; BB, Beta-blocker; CAA, central alpha agonist; APA, alpha-1 peripherally acting antagonist; ARA, aldosterone receptor antagonist; KSP, Potassium-sparing diuretics; FDC, Fixed-dose combination; CI, confidence interval.

### 3.5 Subgroup analyses

Subgroup analyses among individuals with compelling indications are presented in Table 5. Because multiple subgroup-by-class comparisons were performed, we controlled for the FDR using the Benjamini–Hochberg procedure and report FDR-adjusted q-values. After FDR correction, ARB use increased post-guidelines among patients with diabetes (OR = 1.27, 95% CI: 1.07–1.51, q = 0.0383) and stroke (OR = 1.53, 95% CI: 1.14–2.05, q = 0.0361). CCB utilization increased post-guidelines among Non-Hispanic Black patients (OR = 1.47, 95% CI: 1.22–1.77, q = 0.0031).

**TABLE 5:** Impact of 2017 ACC/AHA guidelines implementation on the antihypertensive classes utilization in the subgroups.

Antihypertensive class	Odds ratio of antihypertensive utilization post-2017 ACC/AHA guidelines	Lower 95% CI	Upper 95% CI	q-value
Patients with diabetes				
ACEI	1.05	0.89	1.24	0.7643
ARB	1.27	1.07	1.51	0.0383
CCB	1.19	1.01	1.41	0.1565
Diuretic	1.05	0.89	1.24	0.7752
BB	1.00	0.84	1.20	0.9963
CAA	0.79	0.56	1.12	0.3932
APA	0.88	0.57	1.34	0.7643
ARA	1.42	0.98	2.07	0.2157
Vasodilator	0.99	0.64	1.52	0.9964
FDC	0.68	0.55	0.85	0.0121
Patients with chronic kidney disease				
ACEI	0.86	0.46	1.63	0.8425
ARB	0.91	0.49	1.67	0.9338
CCB	1.31	0.76	2.27	0.5840
Diuretic	0.72	0.43	1.21	0.4935
BB	0.89	0.46	1.71	0.9332
CAA	0.96	0.46	1.99	0.9964
APA	0.34	0.13	0.88	0.1501
ARA	8.29	2.31	29.79	0.0281
Vasodilator	1.83	0.64	5.28	0.4792
FDC	0.58	0.28	1.21	0.4241
Patients with heart failure				
ACEI	0.78	0.48	1.27	0.5839
ARB	1.56	1.02	2.28	0.2393
CCB	1.06	0.69	1.63	0.9803
Diuretic	1.51	0.92	2.44	0.2897
BB	1.00	0.57	1.77	0.9964
CAA	0.42	0.13	1.39	0.4199
APA	0.67	0.06	7.53	0.9332
ARA	2.60	1.51	4.47	0.0378
Vasodilator	0.68	0.37	1.24	0.5839
FDC	0.27	0.10	0.73	0.0752
Patients with myocardial infarction				
ACEI	0.69	0.53	0.91	0.0394
ARB	1.26	0.94	1.68	0.2897
CCB	1.08	0.81	1.45	0.7910
Diuretic	0.89	0.68	1.16	0.5839
BB	1.48	1.08	2.03	0.0752
CAA	1.04	0.58	1.89	0.9868
APA	1.03	0.57	1.83	0.9963
ARA	1.18	0.80	1.73	0.6064
Vasodilator	0.66	0.40	1.09	0.2738
FDC	0.76	0.52	1.10	0.3153
Non-hispanic black				
ACEI	0.76	0.62	0.93	0.0383
ARB	1.34	1.06	1.71	0.0751
CCB	1.47	1.22	1.77	0.0031
Diuretic	1.15	0.94	1.42	0.3770
BB	0.98	0.81	1.19	0.9868
CAA	0.52	0.37	0.73	0.0061
APA	0.79	0.47	1.31	0.5840
ARA	0.96	0.64	1.43	0.9868
Vasodilator	0.70	0.47	1.04	0.2393
FDC	0.71	0.56	0.88	0.0281
Patients with coronary heart disease				
ACEI	0.80	0.64	1.01	0.2143
ARB	1.29	0.99	1.70	0.2143
CCB	1.00	0.78	1.28	0.9964
Diuretic	0.99	0.77	1.28	0.9964
BB	1.26	0.95	1.68	0.2738
CAA	0.73	0.45	1.19	0.4029
APA	0.65	0.37	1.12	0.2897
ARA	1.04	0.66	1.64	0.9868
Vasodilator	0.78	0.47	1.29	0.5640
FDC	0.58	0.40	0.84	0.0347
Patients with stroke				
ACEI	0.88	0.67	1.16	0.5839
ARB	1.53	1.14	2.05	0.0361
CCB	1.15	0.91	1.44	0.4585
Diuretic	1.27	0.93	1.74	0.2897
BB	1.24	0.96	1.62	0.2738
CAA	0.46	0.27	0.81	0.0383
APA	1.15	0.65	2.01	0.8358
ARA	1.69	1.00	2.85	0.1858
Vasodilator	0.96	0.54	1.70	0.9868
FDC	0.57	0.38	0.85	0.0291

Abbreviation: ACC, american college of cardiology; AHA, american heart association; ACEI, Angiotensin-converting enzyme inhibitors; ARB, angiotensin receptor blocker; CCB, calcium channel blocker; BB, Beta-blocker; CAA, central alpha agonist; APA, alpha-1 peripherally acting antagonist; ARA, aldosterone receptor antagonist; KSP, Potassium-sparing diuretics; FDC, Fixed-dose combination; CI, confidence interval.

FDC use decreased across majority of subgroups, including those with diabetes (OR = 0.68, 95% CI: 0.55–0.85, q = 0.0121), stroke (OR = 0.57, 95% CI: 0.38–0.85, q = 0.0291), coronary heart disease (OR = 0.58, 95% CI: 0.40–0.84, q = 0.0347), and Non-Hispanic Black patients (OR = 0.71, 95% CI: 0.56–0.88, q = 0.0281). Moreover, ACEI use declined in Non-Hispanic Black patients (OR = 0.76, 95% CI: 0.62–0.93, q = 0.0383) and patients with myocardial infarction (OR = 0.69, 95% CI: 0.53–0.91, q = 0.0394). ARA use increased among CKD patients (OR = 8.29, 95% CI: 2.31–29.79, q = 0.0281) and heart failure patients (OR = 2.60, 95% CI: 1.51–4.47, q = 0.0378).

## 4 Discussion

Despite major changes in HTN guidelines over the past decade, national trends in antihypertensive prescribing patterns, particularly following the 2017 ACC/AHA guidelines update, remain uncharacterized. Most post-guidelines studies have focused on HTN prevalence, control, or treatment eligibility, rather than longitudinal changes in medication utilization. Furthermore, the existing evidence on antihypertensive use after the 2017 ACC/AHA guidelines has relied on data only through 2019 from non–nationally representative cohorts, leaving a gap in understanding long-term, population-level prescribing patterns (Dorans et al., 2018; Muntner et al., 2018; Khera et al., 2018; Hussain et al., 2022; Lin et al., 2024). To address this, we conducted a nationally representative analysis using MEPS data from 2013 to 2022 to evaluate changes in antihypertensive utilization before and after the 2017 ACC/AHA guidelines release. The results showed significant changes in antihypertensive prescribing patterns following the guidelines update. The use of ARB and CCB increased, whereas the use of FDC therapies declined. Other drug classes showed minimal but not statistically significant change. These findings suggest a partial alignment between real-world prescribing and guidelines recommendations, while also highlighting a persistent gap in the optimal management of HTN.

Following the 2017 ACC/AHA guidelines update, ARB use increased significantly among US adults with treated HTN. This trend reflects the evolving clinical practice shaped by ARB efficacy, tolerability, and accessibility. Although both ACEI and ARB are recommended as first-line agents, ARB are associated with a lower incidence of cough and angioedema, adverse effects that frequently lead to ACEI discontinuation (Whelton et al., 2017; Chen et al., 2021; Li et al., 2014). This favorable safety profile may have contributed to ARB being increasingly prescribed, particularly for patients with prior ACEI intolerance or when clinicians anticipate adherence challenges. A recent observational study supports this trend. Lin et al. (2024), analyzing large EHR datasets, demonstrated a significant post-2017 ACC/AHA guidelines rise in ARB prescriptions, concurrent with a decline in ACEI use in hypertensive patients (Lin et al., 2024). Cost and accessibility factors may also explain this trend, as numerous ARB agents became available in generic forms after patent expirations, reducing financial barriers for both patients and payers (Cooper-DeHoff and Elliott, 2013). Beyond tolerability and affordability, ARB provides BP-lowering efficacy comparable to ACEI, with accumulating evidence supporting similar cardiovascular and renal protective effects (Chen et al., 2021).

In parallel, we observed an increase in CCB utilization following the 2017 ACC/AHA guidelines update. This finding is consistent with prior observational studies reporting increased CCB prescribing in the post-guidelines era (Lin et al., 2024). CCB has long been recommended as a first-line agent for the Non-Hispanic Black population in both the JNC 8 and 2017 ACC/AHA guidelines, given their robust evidence of BP-lowering efficacy and favorable cardiovascular outcomes (James et al., 2014; Whelton et al., 2017). The broad availability of long-acting, generic CCBs such as amlodipine has enhanced affordability and accessibility, likely contributing to the increased use (Jacobs et al., 2024). Furthermore, CCBs are generally well tolerated, offer convenient once-daily dosing, and have low discontinuation rates, making them attractive options for long-term HTN management (Koracevic et al., 2015). The observed increase in CCB prescribing may also reflect the clinician’s preference for agents with fewer metabolic adverse effects, particularly among patients with diabetes or dyslipidemia risk (Whelton et al., 2017).

Major HTN guidelines, including the 2017 ACC/AHA and European Society of Cardiology/European Society of HTN, advocate FDC use as initial or early add-on therapy, due to strong evidence that FDC improves medication adherence by 15%, simplifies regimens, and is associated with better BP control and improved cardiovascular outcomes (Whelton et al., 2017; McEvoy et al., 2024; Du et al., 2018). Despite this, our study showed a significant decline in FDC utilization following the 2017 ACC/AHA guideline, representing a concerning gap between the recommended guidelines and real-world practice (Whelton et al., 2017). Consistent with our findings, a recent study reported a reduction in FDC antihypertensive utilization from 11% to 9% in 2018–2019, compared with the 2015–2017 period (Lin et al., 2024). Similarly, a recent nationally representative analysis demonstrated a decline in FDC antihypertensives use between 2009 and 2020 from 35.8% to 25.8% (Mobley et al., 2023). Some barriers may explain this decline in FDC use. One of the barriers is the prescribers’ preference for flexible, individualized dose titration and treatment intensification; clinicians often favor separate agents so each drug can be adjusted independently, which is not always feasible with available FDC. Clinical inertia may further limit FDC adoption, especially among patients already stable on multi-pill regimens. Additionally, a lack of familiarity with the growing number of FDC options may limit prescribing FDC (Essa et al., 2024). Finally, some prescribers are concerned about distinguishing side effects when prescribing FDC (Essa et al., 2024).

Beyond clinician-level factors, several health system and policy barriers likely contribute to the decline in FDC prescribing. Formulary restrictions and incomplete inclusion of many antihypertensive FDCs on public and private formularies can limit access to guideline-preferred combinations or specific dose strengths, and in some cases, place them on higher cost-sharing tiers (O'Hagan et al., 2024). Insurance coverage policies and prior authorization requirements may further discourage routine FDC use when separate agents are more easily approved (An et al., 2020). Additionally, although several FDCs are now available as generics, many clinically preferred combinations and strengths are still not commercially available, which constrains prescribers’ ability to align real-world regimens with guideline-recommended single-pill combinations (An et al., 2020). These system-level barriers, together with clinician preferences and treatment inertia, likely contribute to the declining FDC use we observed despite strong evidence and guideline support for their role in improving adherence and BP control.

Subgroup analyses revealed prescribing patterns largely concordant with guidelines recommendations and established clinical evidence. Increased CCB use among non-Hispanic Black adults aligns with evidence demonstrating superior BP reduction and cardiovascular outcomes in this population (Whelton et al., 2017; Officers, 2002). Similarly, increased ARB utilization among individuals with diabetes and stroke reflects the translation of evidence-based, organ-protective strategies into routine practice, as recommended by the guidelines (Whelton et al., 2017). The decline in ACEI use among Black adults is both guideline-concordant and clinically appropriate, acknowledging the lower efficacy of ACEI and increased risk of cough and angioedema in this population (Whelton et al., 2017; Ogedegbe et al., 2015; Reichman et al., 2017). In addition, the increased use of ARA among CKD and heart failure patients demonstrates greater adoption of therapies proven to reduce morbidity and mortality in these patients (Pitt et al., 1999; Zannad et al., 2011; Bakris et al., 2020; Pitt et al., 2021). FDC use decreased across the majority of the subgroups. Collectively, these subgroup findings highlight encouraging improvements in adherence with guidelines recommendations, but also reveal a persistent gap, particularly the underuse of FDC.

These findings have important implications for clinical practice and health policy. The increased use of ARB and CCB suggests meaningful progress toward implementing evidence-based care that is likely to enhance BP control and cardiovascular risk reduction. However, the persistent decline in FDC antihypertensive utilization across the cohort signals a missed opportunity to improve medication adherence and BP control, especially in those with multiple comorbidities, who require multi-drug therapeutic strategies and are at greater risk of adverse cardiovascular outcomes. Given the strong evidence supporting FDC for simplifying therapy and improving clinical outcomes, this trend emphasizes the need for targeted provider education and health system interventions to promote appropriate FDC use. Further research and policy efforts are needed to identify and address barriers to FDC adoption.

This study has some limitations that warrant consideration. First, antihypertensive medication use was identified through self-report and prescription fill data; although MEPS includes validated pharmacy records, prescription fills do not guarantee medication adherence, potentially leading to misclassification of true drug exposure. Second, the dataset offers limited insight into the clinical rationale behind prescribing decisions, such as intolerance, medication cost, or formulary availability, making it challenging to interpret prescribing shifts. Third, we did not distinguish between incident and prevalent HTN or account for baseline BP severity or timing of treatment initiation, which may influence medication selection. Also, as MEPS lacks BP measurements to allow for stage 1 and stage 2 HTN classification, we could not determine whether the observed increase in ARB and CCB use occurred among patients with stage 1 HTN newly targeted for treatment under the 2017 guideline. Fourth, while major comorbidities such as diabetes, heart failure, and CKD were captured, MEPS lacks granular clinical detail (e.g., left ventricular ejection fraction, proteinuria, or stroke subtype), limiting the ability to assess appropriateness of therapy relative to guideline-based indications. Fifth, MEPS does not contain a complete list of FDC products. Sixth, the observed post-guidelines increase in antihypertensives utilization may reflect changes in prescriber behavior, a larger pool of patients qualifying for therapy, or both, after the 2017 ACC/AHA guidelines lowered diagnostic and treatment threshold. Lastly, despite robust multivariable adjustment, unmeasured confounding, such as provider preference, patient preferences, or institutional prescribing policies, may still bias the associations observed. Despite these limitations, this study offers several notable strengths. The study provided a nationally representative assessment of antihypertensive prescribing patterns among US adults with treated HTN. The decade-long study period allowed for a robust pre- and post-guidelines comparison that captures changes in prescribing behavior following the 2017 ACC/AHA HTN guidelines update. Inclusion of a broad range of antihypertensive classes and clinically relevant subgroups enabled a comprehensive assessment of real-world prescribing patterns.

## 5 Conclusion

Our study showed both encouraging progress and persistent gaps in antihypertensive prescribing following the release of 2017 ACC/AHA HTN guidelines. The observed increase in ARB and CCB use reflects progress toward more evidence-based HTN management. In contrast, the decline in FDC utilization in the cohort represents a missed opportunity for improving adherence, BP control, and ultimately cardiovascular outcomes, especially in patients with multiple comorbidities. These findings highlight the need for enhanced provider education, targeted health system interventions, and supportive policy efforts to bridge the gap between clinical guidelines and real-world practice.

## Data Availability

The datasets presented in this study can be found in online repositories. The names of the repository/repositories and accession number(s) can be found below: https://meps.ahrq.gov/mepsweb/data_stats/download_data_files.jsp.
