# Supplementary material for: Impact of the 2017 ACC/AHA hypertension guidelines on antihypertensive prescribing in the United States: real-world evidence from a nationally representative population
Source: Front Pharmacol. 2026 Feb 25;17:1787466. doi: 10.3389/fphar.2026.1787466 (PMC12976020; doi:10.3389/fphar.2026.1787466)
Supplement: Supplementary file 1 [file Table1.docx]

Supplementary Materials

Table S1. Antihypertensives Multum Lexicon therapeutic classification codes

| Therapeutic classification codes | Drug class |
| --- | --- |
| TC1S1 = 55 | Antihypertensive combinations |
| TC1S1 = 49 | Diuretics |
| TC1S1 = 42 | Angiotensin converting enzyme inhibitors |
| TC1S1 = 47 | Beta-adrenergic blocking agents |
| TC1S1 = 56 | Angiotensin II inhibitors |
| TC1S1 = 48 | Calcium channel blocking agents |
| TC1S1 = 53 | Vasodilators |
| TC1S1 = 44 | Antiadrenergic agents, centrally acting |
| TC1S1 = 43 | Antiadrenergic agents, peripherally acting |
| TC1S1_1 = 156 | Thiazide diuretics |
| TC1S1_1 = 475 | Potassium-sparing diuretics with thiazide |
| TC1S1_1 = 479 | Angiotensin II inhibitors with calcium ch |
| TC1S1_1 = 476 | ACE inhibitors with calcium channel blockers |
| TC1S1_1 = 473 | Angiotensin II inhibitors with thiazides |
| TC1S1_1 = 472 | Beta blockers with thiazides |
| TC1S1_1 = 470 | Miscellaneous antihypertensive combination |
| TC1S1_1 = 467 | ACE inhibitors with thiazides |
| TC1S1_1 = 154 | Loop diuretics |
| TC1S1_1 = 155 | Potassium-sparing diuretics |
| TC1S2 = 340 | Aldosterone receptor antagonists |
